# New Books

**Published:** 1874-02

**Authors:** 


New Books.—Books are a necessity to a reading people. No man can meet the demands of society unless he shall inform himself of the current literature of the day. If he is in a position where he is expected to know something of science, and to keep pace with it, he must read scientific periodicals and scientific books. If he is a physician his taste, his inclination and his duty to his patients, all should demand that he keep himself up with the science of medicine. He can do this only by securing the best medical literature to be found in periodicals and books. With the object of bringing before our readers new works in the various departments of the healing art, we shall, from month to month, give them such notices of books as shall be sent us. We shall carefully note their excellencies or want of merit, and give our readers the benefit of a candid opinion.

We have been called upon to furnish lists of such books as we could recommend, and have done so, we trust with satisfaction. We will always aid our friends in making selections for their libraries when desired to do so, and as they may repose confidence in our judgment rather than to rely upon the catalogues of publishers, we shall give them the full benefit of our knowledge, and only advise the purchase of those we can honestly recommend.

